# *Limosilactobacillus reuteri* FN041 prevents atopic dermatitis in pup mice by remodeling the ileal microbiota and regulating gene expression in Peyer’s patches after vertical transmission

**DOI:** 10.3389/fnut.2022.987400

**Published:** 2022-09-28

**Authors:** Jingbo Zhou, Gaoshun Xu, Xinyue Li, Huayu Tu, Haoyu Li, Hong Chang, Jie Chen, Renqiang Yu, Ce Qi, Jin Sun

**Affiliations:** ^1^Institute of Nutrition and Health, Qingdao University, Qingdao, China; ^2^Department of Pediatric Cardiology Nephrology and Rheumatism, The Affiliated Hospital of Qingdao University Medical College, Qingdao, China; ^3^Department of Neonatology, The Affiliated Wuxi Maternity and Child Health Care Hospital of Nanjing Medical University, Wuxi, China

**Keywords:** *Limosilactobacillus reuteri*, atopic dermatitis, vertical transmission, regulatory T cells, ileal mucosal barrier, Peyer’s patches, retinol metabolism

## Abstract

**Objectives:**

*Limosilactobacillus reuteri* FN041 is a potential probiotic bacterium isolated from breast milk in traditional farming and pastoral areas of China. The purpose of this study was to investigate the optimal intervention mode and potential mechanism of FN041 to prevent atopic dermatitis (AD) in mice.

**Methods:**

In intervention mode I, FN041 was supplemented to dams during the late trimester and lactation and pups after weaning; in intervention mode II, FN041 was supplemented after pups were weaned. AD was induced in pups with MC903 plus ovalbumin on the ear after weaning.

**Results:**

The effect of intervention mode I in preventing AD was significantly better than that of intervention mode II. Compared with the model group, the inflammatory response of the pup’s ears, the proportion of spleen regulatory T cells and the plasma IgE were significantly decreased in mice in intervention mode I. Furthermore, the intestinal mucosal barrier was enhanced, and the Shannon index of the ileal microbiota was significantly increased. The microbiota structure deviated from the AD controls and shifted toward the healthy controls according to the PCoA of unweighted UniFrac. The relative abundances of *Limosilactobacillus*, *Faecalibacterium*, *Bifidobacterium*, and *Akkermansia* in the ileum were significantly increased compared to the AD group. Based on RNA-seq analysis of pups’ Peyer’s patches (PPs), FN041 inhibits autoimmune pathways such as asthma and systemic lupus erythematosus and activates retinol metabolism and PPAR signaling pathways to reduce inflammatory responses. Intervention mode II also significantly reduced AD severity score, but the reduction was approximately 67% of that of intervention mode I. This may be related to its ineffective remodeling of the ileal microbiota.

**Conclusion:**

Prenatal and postnatal administration of FN041 is an effective way to prevent AD in offspring, and its mechanism is related to remodeling of ileal microbiota and PPs immune response.

## Introduction

Atopic dermatitis (AD) is a recurrent chronic inflammatory skin disease with the highest incidence in childhood. It is characterized by recurrent chronic eczema-like rashes with dry, itchy and red skin (1). The immunological features are increased systemic allergen-specific IgE production and imbalanced T-cell responses, with impaired immune tolerance being the underlying cause of AD (2). More than one-third of the immune cells in the human body are located in Peyer’s patches (PPs) (3). Antigen transport through PPs is an important factor in the induction of immune tolerance by mucosal and systemic immune defenses. Oral probiotics can interact with PPs, and probiotics coated by secretory immunoglobulin A (sIgA) can be transported into PPs mediated by the sIgA receptor expressed by M cells to induce immune tolerance (4–6). We previously isolated a sIgA-coated potential probiotic strain, *Limosilactobacillus reuteri* FN041, from the breast milk of a healthy mother in southern Gansu. We have found that FN041 supplementation in lactating female mice can be vertically transmitted into breast milk (7). Supplementation of FN041 in female mice during lactation can also regulate the expression of IgA pathway genes in offspring PPs (8, 9). Breast milk is rich in sIgA, and FN041 ingested by the mother interacts with sIgA to modulate the PPs immune response (10). The incomplete development of the intestinal mucosal immune system and low levels of intestinal sIgA in pups prevented effective coating of exogenous probiotics and may have limited probiotic regulation of the PPs immune response (11). It remains to be studied whether there is a difference in the efficiency of preventing AD by supplementing the mothers with FN041 or directly supplementing the offspring with FN041 early in life.

The ileum is the main distribution site of PPs (12). The development of PPs is influenced by the outer environment in the form of nutrition and ileal commensal bacteria (13). Supplementation with FN041 in mice had no significant effect on the hindgut microbiota (7). However, they would colonize in the small intestine and affect the ileal microbiota. A healthy immune system is usually in a tolerant or quiescent state, unresponsive to self-antigens, dietary antigens and commensal bacteria (14). The development of oral tolerance early in life inhibits the production of Th2-type cytokines and suppresses allergy. CD103^+^ dendritic cells in PPs mediate oral immune tolerance through induction of regulatory T cells (15). It metabolizes vitamin A to produce retinoic acid (RA), which is the key to initiating this reaction (16, 17). Whether FN041 would activate the immune tolerance pathway remains to be studied.

The purpose of this study was to investigate the best intervention method of FN041 in preventing AD in mice and its effect on the gene expression profile of ileal microflora and PPs in AD mice and to provide evidence for the use of FN041 in preventing allergic diseases in infants.

## Materials and methods

### Materials

Calcipotriene (MC903) was purchased from Sigma-Aldrich (St. Louis, MO, USA). Mast cell staining solution was purchased from Sembega Biotechnology Co., Ltd., (Nanjing, China). Ovum albumin (OVA) was purchased from Thermo Fisher Scientific Co., Ltd., (USA). Hematoxylin-Eosin (H&E) staining solution was purchased from Biyuntian Biotechnology Co., Ltd., (Shanghai, China). Enzyme-linked immunosorbent assay (ELISA) kits for IL-12, IL-33, IL-10, IL-4, IgE, thymic stromal lymphopoietin (TSLP), zonulin, IgG1, IgG2a and sIgA were purchased from JingMei Biotechnology Co., Ltd., (Suzhou, China). MULTI SCIENCES Co., Ltd., (Hangzhou, China) provided the Mouse Treg staining Kit.

### Animal experiments

Qingdao University Animal Ethics Committee approved the animal experimentation procedure (No. 20190118) and was carried out based on the National Guidelines for Experimental Animal Welfare. Seven-week-old male, female and three-week-old BALB/C mice (SPF grade) were purchased from SPF Biotechnology Co., Ltd., (Beijing, China) and fed a chow-based diet *ad libitum* to the mice.

**FIGURE 1:**
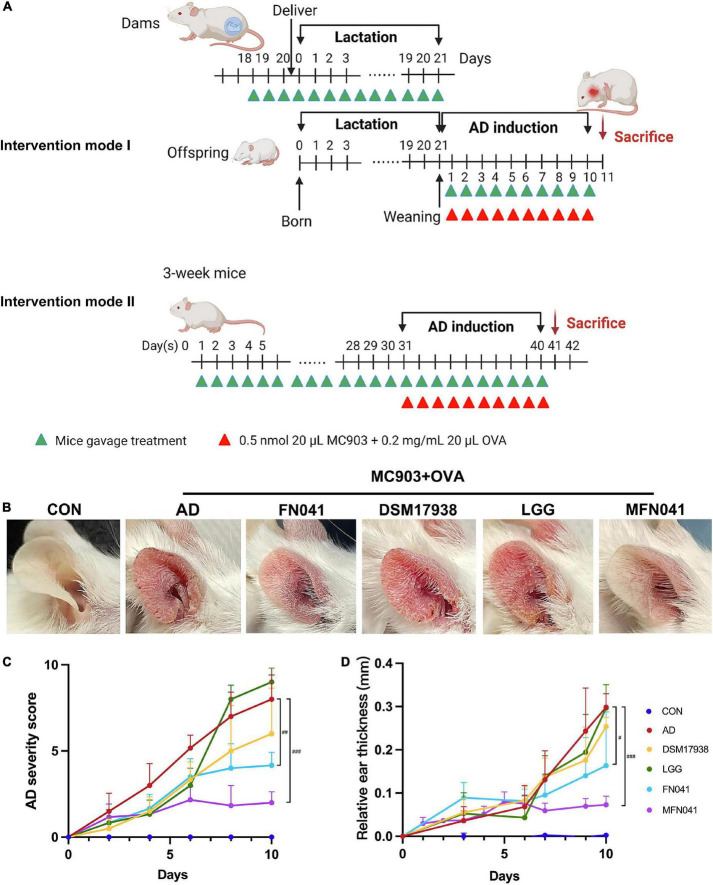
Effect of different intervention modes on MC903 plus OVA-induced symptoms of atopic dermatitis in mice. **(A)** Procedure for two intervention modes. **(B)** Gross appearances of the ears on day 10 after AD induction. **(C)** AD severity score, including the number of scratching bouts, erythema/hemorrhage, eruption, edema and scaling-like changes in the ear on a scale of 0–10, with higher scores indicating more severe AD symptoms (*n* = 12). **(D)** Ear thickness relative to that of day 0 after MC903 plus OVA induction (*n* = 12). #*P* < 0.05, ##*P* < 0.01, ###*P* < 0.001 vs. AD group (one-way ANOVA test). CON, normal control mice supplemented with sanitary saline; AD, supplementation with sanitary saline after weaning of pups before AD induction; DSM17938, supplementation with DSM17938 after weaning of pups before AD induction; LGG, supplementation with LGG after weaning of pups before AD induction; FN041, supplementation with FN041 after weaning of pups before AD induction; MFN041, FN041 supplementation in dams during the late trimester and lactation period and after weaning of pups before AD induction.

The mice were maintained in an environment with a controlled light cycle (12 h light/12 h darkness), humidity level (40 – 50%) and temperature (20 – 25°C). There are two intervention modes. For intervention mode I, female and male mice were caged under sanitary conditions overnight at a female/male ratio of 2/1 and examined for a vaginal plug in the next morning. After the females were impregnated, the males were removed from the cages. Maternal mice supplemented with FN041 (1 × 10^9^ CFU/day, 100 μl once a day) started from the 3rd day before parturition until the third week of lactation (*n* = 6). Then the pups were supplemented with FN041 (1 × 10^9^CFU/day, 100 μl once a day) until day 10. Pups were induced with 20 μl of 0.05 nmol MC903 plus 20 μl of 0.2 mg/ml OVA applied to the right ear and anhydrous ethanol to the left ear for AD induction until day 10 (18). Then pups were euthanized on day 11 (*n* = 12; MFN041 group). For intervention mode II, sixty 3-week-old BALB/C pups were randomly divided into five groups (*n* = 12), normal control (CON) group (supplemented with sanitary saline), atopic dermatitis (AD) group (supplemented with sanitary saline), DSM17938 group (supplemented with *Limosilactobacillus reuteri* DSM 17938), LGG group (supplemented with *Lacticaseibacillus rhamnosus* GG), FN041 group (supplemented with *Limosilactobacillus reuteri* FN041) (1 × 10^9^ CFU/day, 100 μl once a day). Supplementation was lasted for 40 days to the pups. In addition to the CON group, AD was induced on days 31 – 40, and mice were euthanized on day 41. During AD induction, the thickness of the right ear was measured and the severity of AD was scored every day. Supplementary materials provide details of scoring criteria. Mice were anesthetized and sacrificed at the end of the experiment, and various tissues were collected. The experimental procedure is shown in Figure 1A.

### Histological examination

Ears were fixed in 4% phosphate-buffered paraformaldehyde. Inflammatory cell infiltration was assessed for H&E stained section (19, 20). Section was also strained with toluidine blue to detect mast cells, while Carbol 2R hematoxylin was used to detect eosinophils. The number of cells in 10 random fields of view of the ear section was calculated. Villi height and crypt depth of mice ileum were calculated from 10 random fields of view. Supplementary materials provide details.

### Flow cytometric analyses

Spleens were placed in a 15 ml polypropylene tube on ice (no liquid) and 5 ml red blood cell (RBC) lysis buffer was added. Spleens were then smashed thoroughly on the rough side of the frosted part of the slide (wetted with RBC lysis buffer) on a 10 cm tissue culture dish to dissociate cells.

Splenocytes were transferred into a 15 ml tube and 10 ml of RPMI1640 medium was added to invert 2–3 times. After centrifugation at 1,300 rpm and 4°C for 5 min, the pellet was resuspended in 3 ml RPMI1640 medium and filtered through a 70 μM filter over a new 50 ml conical tube. Cells were diluted to 1 × 10^7^ cells/ml and were stained with FITC-labeled CD4^+^ and APC-labeled CD25^+^ monoclonal antibodies, respectively. Red blood cell lysate and flow staining buffer were added, and the supernatant was discarded after centrifugation at 400 × *g* for 5 min at room temperature, and the fixative was added to incubate at room temperature for 60 min in the dark. It was centrifuged at 400 × *g* for 5 min at room temperature and resuspended. Cells were stained with PE-labeled Foxp3^+^ antibody, while anti-IgG1 was used as an isotype control. Flow cytometric assay was performed on the BD FACSVerse (BD Biosciences, Franklin Lakes, NJ, USA) or Aurora (Cytek, Fremont, CA, USA), and data were analyzed using the FlowJo 10.0.7 software (Tree Star, Ashland, OR, USA). For each sample, 2 × 10^4^ cells were counted.

### Measurement of the cytokines in plasma and ear of pups

In accordance with the instructions provided by the manufacturer of the ELISA kit, IL-12, IL-10, IL-4, IgE, IgG1, IgG2a, zonulin, sIgA in plasma, IL-33 and TSLP in 10% ear homogenate were determined.

### Analysis of the bacterial community in the ileum

We extracted microbiota from the total ileum contents according to the method previously described (21, 22). As described in the Supplementary materials, 16S rDNA amplicon sequencing of the V3-V4 region was performed on a next-generation sequencing platform using a double-end (Paired-End) method (23). All raw sequencing data were deposited at the NCBI Sequence Read Archive with accession number PRJNA827499.

### RNA sequencing and gene set enrichment analysis

RNA sequencing for PPs as described in Supplementary methods. The R package “limma” was utilized for the normalization of RNA expression profiles. Differentially expressed genes (DEGs) were tested using the bayesian adjusted t-statistics from the linear models of limma. A multiple testing correction based on the false discovery rate (FDR) was performed. Log2 (fold change) (log2FC) > 1.5 and *P* < 0.05 were used as the cut-off criteria of DEGs samples. DEGs that met this criterion were used to perform gene set enrichment analysis for biological process themes of Gene Ontology (GO) and Kyoto Encyclopedia of Genes and Genomes (KEGG) pathway using the R package “Clusterprofiler v4.0” (24). A corrected *P* < 0.05 was the cut-off criterion. KEGG pathways were visualized with Pathview.

### Statistical analyses

Data were expressed as mean ± standard error (SEM) or median [IQR]. SPSS 24 software was used to analyze the data. GraphPad Prism 9 was used to visualize the results. When the data variance was equal, one-way ANOVA and Tukey’s *post hoc* tests were applied, and when it wasn’t, Kruskal-Wallis tests were applied. False discovery rate (FDR) corrected *P*-values are applied when appropriate. The alpha diversity analysis of Shannon, Chao1 and Simpson indices was calculated by the R package vegan and evaluated using the Wilcoxon rank-sum test. Differences in the β-diversity were visualized using principal coordinate analysis (PCoA) plots and inferred using the R package multivariate analysis of variance (PERMANOVA) to test for inference. For the constrained ordination approach, constrained principal coordinate analysis (CPCoA) was performed on the Bray–Curtis distance matrix using *capscale* and *anova.cca* functions and the resulting ordination was visualized by *ggplot2*. Correlation analysis and Spearman analysis were performed using the R package psych, and the data matrix was visualized using the R package pheatmap. The *P*-values were adjusted for multiple comparisons using FDR. Differences were considered statistically significant when *P*-value was < 0.05. The relative proportion of PPs immune cells was compared between two groups by two-sided Welch’s *t*-test with Benjamini-Hochberg FDR correction.

## Results

### Eczema was more effectively prevented in mice with intervention mode I

**FIGURE 2:**
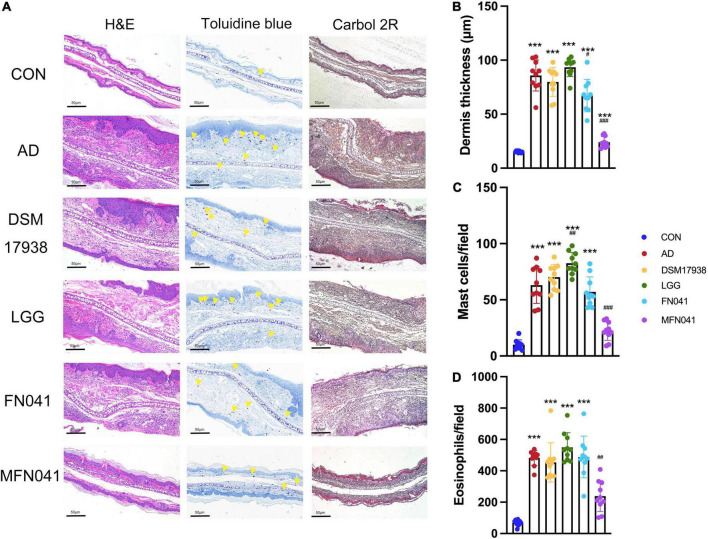
Effect of different intervention modes on ear inflammation in mice with atopic dermatitis. **(A)** Histology of the skin lesions. Hematoxylin and eosin (H&E) staining showed the thickness of the dermis, toluidine blue staining showed the infiltration of mast cells, and Carbol 2R hematoxylin indicated eosinophils. **(B)** H&E staining shows the thickness of the dermis in 10 random fields of view of mice ear sections. **(C)** Mast cells are indicated by yellow triangles on the images, and their numbers represent the number of mast cells in 10 random fields of view of mouse ear sections. **(D)** Eosinophils are the number of cells in 10 random fields of view of mice ear sections. ***P < 0.001 vs. CON group; #*P* < 0.05, ##*P* < 0.01, ###*P* < 0.001 vs. AD group (one-way ANOVA test). CON, normal control mice supplemented with sanitary saline; AD, supplementation with sanitary saline after weaning of pups before AD induction; DSM17938, supplementation with DSM17938 after weaning of pups before AD induction; LGG, supplementation with LGG after weaning of pups before AD induction; FN041, supplementation with FN041 after weaning of pups before AD induction; MFN041, FN041 supplementation in dams during the late trimester and lactation period and after weaning of pups before AD induction.

**FIGURE 3:**
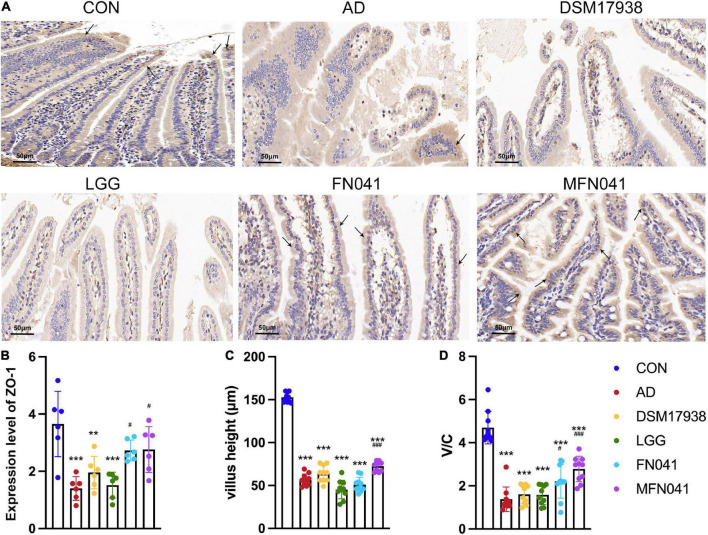
Effects of different intervention modes on the ileal mucosal barrier and histomorphology. **(A)** Immunohistochemistry showing ZO-1 expression (black arrows) in the ileal mucosa of mice in each group. **(B)** The expression level of ZO-1 in the ileum (*n* = 6). **(C)** Mouse ileal villus height expressed as height in 10 random fields of mouse ear slices. **(D)** The ratio of ileal villus height to crypt depth. **P < 0.01, ***P < 0.001 vs. CON group; #*P* < 0.05, ###*P* < 0.001 vs. AD group (one-way ANOVA test). CON, normal control mice supplemented with sanitary saline; AD, supplementation with sanitary saline after weaning of pups before AD induction; DSM17938, supplementation with DSM17938 after weaning of pups before AD induction; LGG, supplementation with LGG after weaning of pups before AD induction; FN041, supplementation with FN041 after weaning of pups before AD induction; MFN041, FN041 supplementation in dams during the late trimester and lactation period and after weaning of pups before AD induction.

On the third day of MC903 plus OVA treatment, the relative thickness of the ears of mice in the AD, DSM17938, LGG, FN041, and MFN041 groups increased significantly compared to the CON group (*P* < 0.05, Figure 1D). On the 10th day of AD induction, except for the MFN041 group, the ear thickness continued to increase in all the probiotic-treated mice with varying degrees of itching, redness, dryness, scaling, and local erosion (Figure 1B). The relative thickness of the ears of mice in the AD group increased by 308.9 and 82.8% compared to the MFN041 and FN041 groups, respectively (Figure 1D). Compared to the AD group, the FN041 group (*P* < 0.01) and the MFN041 group (*P* < 0.001) mice had mild AD symptoms (Figure 1C). Mice in the AD, LGG and DSM17938 groups had swollen epidermis and dermis of the auricle and severe inflammatory cell infiltration. The thickness of the right ear dermis of mice in only the MFN041 (*P* < 0.001) and FN041 (*P* = 0.016) group was significantly thinner than that of the AD group, reduced by 269.1 and 25.8%, respectively (Figures 2A,B). The population of eosinophils and mast cells per unit area of ear tissue was greatly raised in AD mice (*P* < 0.001), and only MFN041 treatment was able to dramatically reduce these symptoms (*P* < 0.01) (Figures 2C,D). The number of mast cells decreased in the FN041 group compared with the AD group, but there was no statistical difference (*P* = 0.083), whereas LGG treatment worsened the situation (*P* = 0.003) (Figure 2C). The expression of ZO-1 in the ileum was significantly lower in the AD (*P* < 0.001), DSM17938 (*P* = 0.002) and LGG (*P* < 0.001) groups compared with the CON group. However, there was no significant difference between the CON group and the FN041 group (*P* = 0.191) and the MFN041 group (*P* = 0.226). It was significantly higher in the FN041 group (*P* = 0.020) and MFN041 group (*P* = 0.016) than in the AD group (Figures 3A,B). The height of ileal villi in the MFN041 group was significantly longer than that in the AD group (*P* < 0.001) (Figure 3C). The ratio of ileal villus height to crypt depth was significantly higher in the FN041 group (*P* = 0.015) and MFN041 group (*P* < 0.001) compared to the AD group, increasing by 60.8 and 102.3%, respectively (Figure 3D).

### FN041 induced splenic Treg proliferation is independent of the intervention mode

**FIGURE 4:**
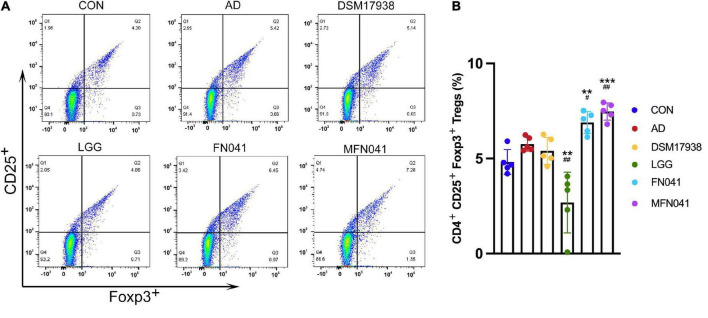
Effect of different intervention modes on regulatory T-cell of spleen of mice with atopic dermatitis induction. **(A)** Isolated cells were obtained from the spleens of different groups of mice. **(B)** Based on the spleen cell percentage, CD4^+^ CD25^+^ Foxp3^+^ cells were calculated. A Kruskal-Wallis analysis was performed between all groups (*n* = 5). **P < 0.01, ***P < 0.001 vs. CON group; #*P* < 0.05, ##*P* < 0.01 vs. AD group. CON, normal control mice supplemented with sanitary saline; AD, supplementation with sanitary saline after weaning of pups before AD induction; DSM17938, supplementation with DSM17938 after weaning of pups before AD induction; LGG, supplementation with LGG after weaning of pups before AD induction; FN041, supplementation with FN041 after weaning of pups before AD induction; MFN041, FN041 supplementation in dams during the late trimester and lactation period and after weaning of pups before AD induction.

Compared with the AD group, Tregs of the spleen was significantly increased in the FN041 group (*P* = 0.028) and MFN041 group (*P* = 0.002), by 15.9 and 22.3%, respectively. It was 56.5% lower in the LGG group than in the AD group (*P* = 0.003) (Figures 4A,B).

### Intervention mode-dependent modulation of cytokines and antibodies by FN041

**FIGURE 5:**
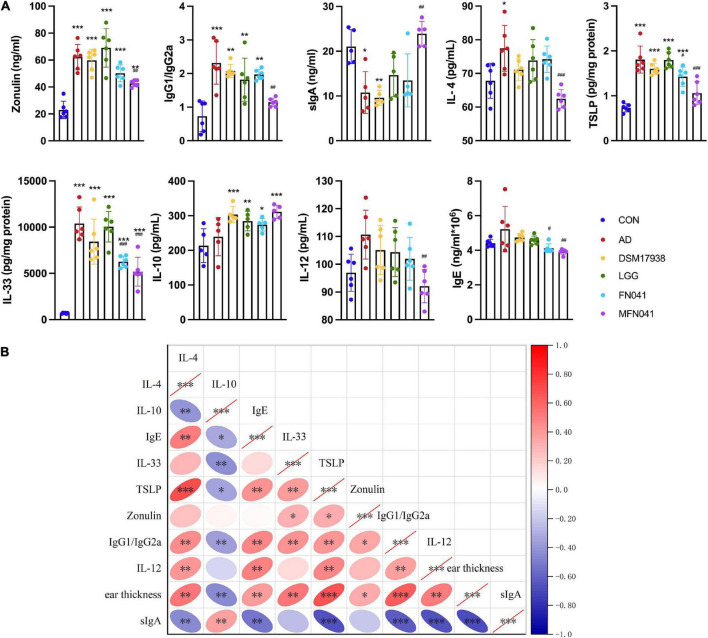
Cytokine levels in mice plasma and ear tissue. **(A)** Zonulin, IL-12, IgG1/IgG2a, IL-4, sIgA, IL-10, IgE in plasma and TSLP, IL-33 in ear tissue. *P < 0.05, **P < 0.01, ***P < 0.001 vs. CON group; #*P* < 0.05, ##*P* < 0.01, ###*P* < 0.001 vs. AD group (one-way ANOVA test) (*n* = 5–6). **(B)** Spearman correlation analysis was performed for cytokines in mice, with red indicating positive correlation and blue indicating negative correlation, with darker colors representing stronger correlation. The right slope of the ellipse represents a negative correlation, and the left slope represents a positive correlation. The flatter the ellipse, the more significant it is. *P < 0.05, **P < 0.01, ***P < 0.001. CON, normal control mice supplemented with sanitary saline; AD, supplementation with sanitary saline after weaning of pups before AD induction; DSM17938, supplementation with DSM17938 after weaning of pups before AD induction; LGG, supplementation with LGG after weaning of pups before AD induction; FN041, supplementation with FN041 after weaning of pups before AD induction; MFN041, FN041 supplementation in dams during the late trimester and lactation period and after weaning of pups before AD induction.

Compared with the AD group, plasma OVA-specific IgG1/IgG2a (*P* = 0.002) and IL-4 (*P* < 0.001) were only significantly decreased in the MFN041 group. Ear IL-33 and TSLP levels were significantly increased in the AD group (*P* < 0.001), which were prevented in the MFN041 group (*P* < 0.001) and FN041 group (*P* < 0.05) (Figure 5A). Correlation analysis showed that ear thickness was significantly positively correlated with IL-4 (*r* = 0.534, *P* = 0.002), IgE (*r* = 392, *P* = 0.032), IL-33 (*r* = 0.539, *P* = 0.002), TSLP (*r* = 0.670, *P* < 0.001), Zonulin (*r* = 0.338, *P* = 0.047), IgG1/IgG2a (*r* = 0.636, *P* < 0.001), IL-12 (*r* = 0.505, *P* = 0.004), and significantly negatively correlated with sIgA (*r* = −0.700, *P* < 0.001) and IL-10 (*r* = –0.442, *P* = 0.015) (Figure 5B).

### Intervention mode I altered gut microbiota in pups

**FIGURE 6:**
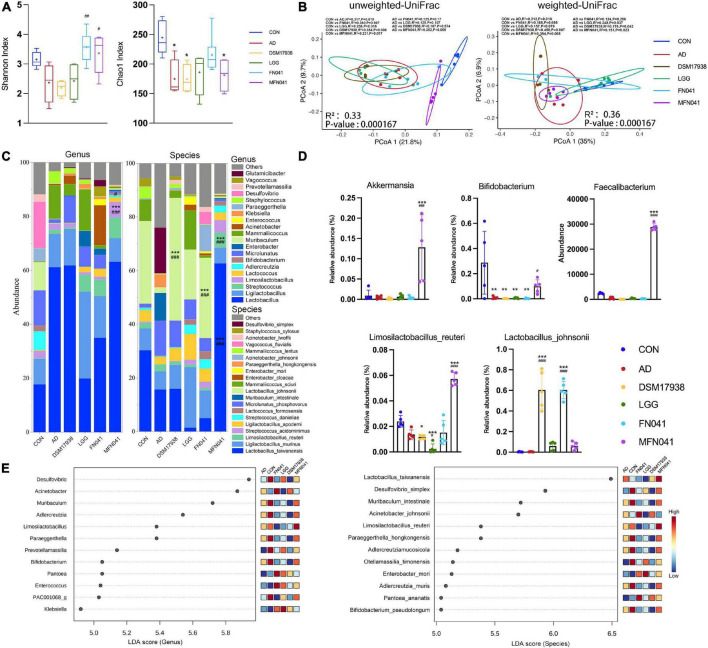
Effect of different intervention modes on ileal microbiota in AD mice. **(A)** Alpha diversity is measured by the chao1 index and Shannon index (*n* = 5). **(B)** Principal coordinate analysis (PCoA) score plots based on unweighted- UniFrac and weighted-UniFrac distance and permutational multivariate analysis of variance (PERMANOVA) was used to test the difference between groups at the OTU level. **(C,D)** Mean relative abundance of genus level and species level. **(E)** Taxa are significantly different between groups based on LEfSe analysis (LDA score > 4). *P < 0.05, **P < 0.01, ***P < 0.001 vs. CON group; #*P* < 0.05, ##*P* < 0.01, ###*P* < 0.001 vs. AD group (*n* = 5). CON, normal control mice supplemented with sanitary saline; AD, supplementation with sanitary saline after weaning of pups before AD induction; DSM17938, supplementation with DSM17938 after weaning of pups before AD induction; LGG, supplementation with LGG after weaning of pups before AD induction; FN041, supplementation with FN041 after weaning of pups before AD induction; MFN041, FN041 supplementation in dams during the late trimester and lactation period and after weaning of pups before AD induction.

**FIGURE 7:**
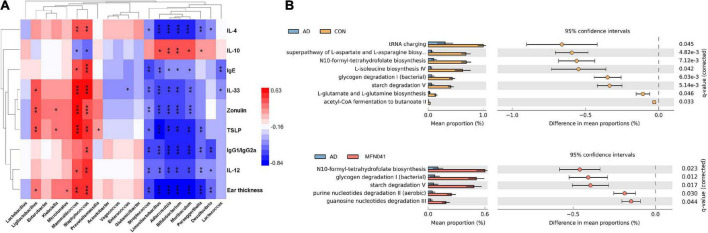
**(A)** Spearman’s correlation coefficients between pro-inflammatory chemokines and cytokines and changes in the relative abundance of individual genera. Positive correlations are shown in red and negative correlations are shown in blue. Darker colors indicate stronger correlations. The False Discovery Rate was used to correct *P*-values for multiple testing. *P < 0.05, **P < 0.01, ***P < 0.001. **(B)** Gene prediction of intestinal flora function of mice (*n* = 5). CON, normal control mice supplemented with sanitary saline; AD, supplementation with sanitary saline after weaning of pups before AD induction; DSM17938, supplementation with DSM17938 after weaning of pups before AD induction; LGG, supplementation with LGG after weaning of pups before AD induction; FN041, supplementation with FN041 after weaning of pups before AD induction; MFN041, FN041 supplementation in dams during the late trimester and lactation period and after weaning of pups before AD induction.

The 16S rDNA amplicon sequencing of mice’s ileal microbiota (v3-v4) was performed. Only for the FN041 (*P* = 0.008) and MFN041 (*P* = 0.038) treatments, the Shannon diversity of the ileal microbiota was elevated by 50.6 and 42.1% as compared to the AD group. The Chao1 was dramatically reduced by AD induction (*P* = 0.009), which was not restored by any probiotic treatment (Figure 6A). According to PCoA, the ileal microbiota composition in the CON group compared to the AD group (*r^2^* = 0.317, *P* = 0.010), the DSM17938 (*r*^2^ = 0.354, *P* = 0.008), LGG (*r^2^* = 0.236, *P* = 0.016), FN041 (*r*^2^ = 0.360, *P* = 0.007) and MFN041 (*r^2^* = 0.231, *P* = 0.017) groups were significantly separated. PCoA of unweighted-UniFrac shows that the flora structure of the MFN041 group was significantly biased toward the CON group (*r^2^* = 0.231, *P* = 0.017) but significantly away from the AD group (*r^2^* = 0.252, *P* = 0.006) (Figure 6B). At the phylum level, the relative abundance of *Tenericutes* was significantly higher in the CON group than that of all other groups (*P* < 0.001). At the genus level, the abundance of *Akkermansia*, *Limosilactobacillus*, and *Faecalibacterium was* significantly higher in the MFN041 group compared to the CON and AD groups (*P* < 0.001). *Bifidobacterium* in the ileum of all AD mice was significantly reduced (*P* < 0.01), except for the MFN041 group (*P* = 0.089) compared to the CON group. At the species level, the abundance of *Lactobacillus taiwanensis* and *Limosilactobacillus reuteri* was significantly increased in the MFN041 group compared to the CON and AD groups (*P* < 0.001). The abundance of *Lactobacillus johnsonii* was significantly increased in the FN041 group and DSM17938 group compared to the AD group (*P* < 0.001) (Figures 6C,D). In addition, linear discriminant analysis (LDA) identified 12 distinct genera spanning six different groups (*P* < 0.05, LDA score > 2.5). The relative abundance of *Desulfovibrio*, *Muribaculum*, and *Bifidobacterium* was higher in the CON group. *Limosilactobacillus*, *Faecalibacterium*, and *Akkermansia* were significantly increased in the MFN041 group (Figures 6D,E). Spearman’s correlation analysis shows that IgE was significantly positively correlated with *Mammaliicoccus* (*r* = 0.457, *P* = 0.011) and *Staphylococcus* (*r* = 0.611, *P* < 0.001), but negatively correlated with *Streptococcus* (*r* = −0.527, *P* = 0.003), *Limosilactobacillus* (*r* = −0.480, *P* = 0.007), *Adlercreutzia* (*r* = −0.385, *P* = 0.036), *Bifidobacterium* (*r* = −0.411, *P* = 0.024), *Muribaculum* (*r* = −0.365, *P* = 0.047), and *Lactococcus* (*r* = −0.473, *P* = 0.008). *Limosilactobacillus* was significantly and positively correlated with IL-10 (*r* = 0.422, *P* = 0.020) but negatively correlated with IL-4 (*r* = −0.637, *P* < 0.001), IgE (*r* = −0.480, *P* = 0.007), IL-33 (*r* = −0.622, *P* < 0.001), Zonulin (*r* = −0.612, *P* < 0.001), TSLP (*r* = −0.760, *P* < 0.001), IgG1/IgG2a (*r* = −0.591, *P* < 0.001), IL-12 (*r* = −0.507, *P* = 0.004) and ear thickness (*r* = −0.811, *P* < 0.001) (Figure 7A).

Functional prediction of ileal microbiota shows that compared with the CON group, tRNA charging (*P* = 0.045), superpathway of L-aspartate and L-asparagine biosynthesis (*P* = 0.004), N10-formyl-tetrahydrofolate biosynthesis (*P* = 0.007), L-isoleucine biosynthesis IV (*P* = 0.042), glycogen degradation I (bacterial) (*P* = 0.006), starch degradation V (*P* = 0.005), L-glutamate and L-glutamine biosynthesis (*P* = 0.004) and acetyl-CoA fermentation to butanoate II (*P* = 0.033) were significantly decreased in the AD group. Compared with the AD group, N10-formyl-tetrahydrofolate biosynthesis (*P* = 0.023), glycogen degradation I (bacterial) (*P* = 0.012), starch degradation V (*P* = 0.017), purine nucleotides degradation II (aerobic) (*P* = 0.030) and guanosine nucleotides degradation III (*P* = 0.044) were significantly increased in the MFN041 group (Figure 7B).

### Intervention mode I regulated gene expression in the Peyer’s patches

**FIGURE 8:**
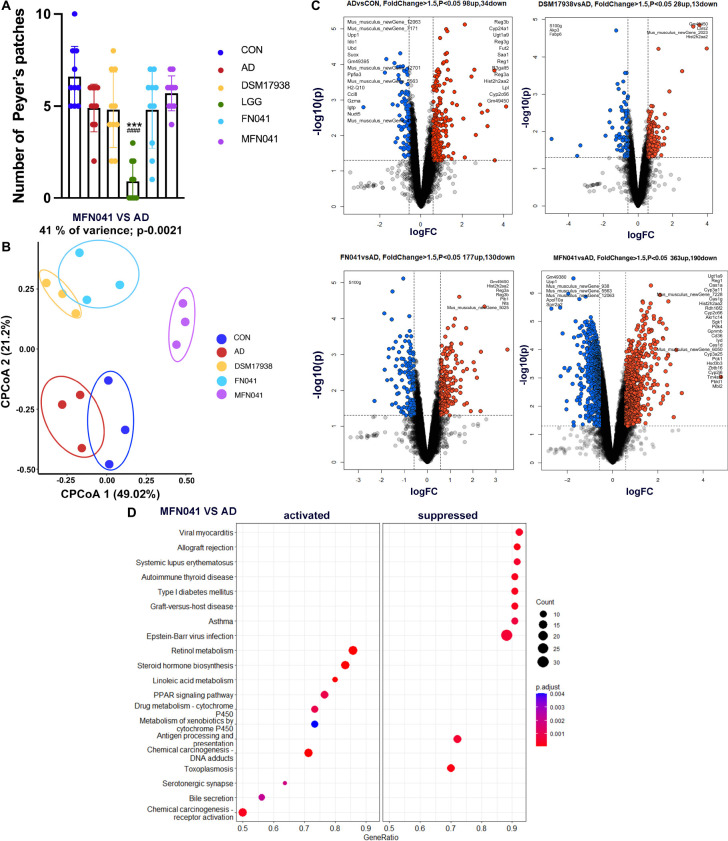
**(A)** The number of PPs (*n* = 10); **(B)** CPCoA, **(C)** Volcano plot, **(D)** KEGG enrichment scatter diagram of differently expressed genes for transcriptome analysis of PPs of offspring mice (*n* = 3). Data are shown as mean ± SD and compared by Student’s *t*-test; ***P < 0.001 vs. CON group; ###*P* < 0.001 vs. AD group. CON, normal control mice supplemented with sanitary saline; AD, supplementation with sanitary saline after weaning of pups before AD induction; DSM17938, supplementation with DSM17938 after weaning of pups before AD induction; LGG, supplementation with LGG after weaning of pups before AD induction; FN041, supplementation with FN041 after weaning of pups before AD induction; MFN041, FN041 supplementation in dams during the late trimester and lactation period and after weaning of pups before AD induction.

We further pooled the PPs of each mouse and performed transcriptome sequencing analysis. Figure 8A shows that the number of PPs in the LGG group was significantly lower than that in the AD group and CON group. The number of PPs in the MFN041 group is 5.33 times of that in the LGG group. The quality of PPs in LGG group mice did not meet the requirements for RNA extraction and transcriptome sequencing could not be performed. The CPCoA results showed that the gene expression profile of the PPs in the MFN041 group of mice was far from the AD group and close to the CON group (Figure 8B). Compared with the CON group, 98 genes were significantly upregulated in the AD group, including Reg3b, Ugt1a9, Hist2h2aa2, etc., (Fold change > 2), and 34 genes were significantly downregulated, including Upp1, Ido1, Suox, H2-Q5, etc., (Fold change < −1). Compared with the AD group, 363 and 190 genes were significantly up- and down-regulated in the MFN041 group, respectively. 23 of the up-regulated genes had Fold change > 2, including Ugt1a9, Cyp3a11, Cyp3a25, Hsd3b3, etc., while the down-regulated genes with Fold change < −2 included Upp1, Apol10a, Sprr2a2, etc. FN041 treatment up-regulated 177 genes including Gm49450, Hist2h2aa2, Reg3a, Reg3b, etc., (Fold change > 2) and down-regulated 130 genes including S100g, etc., (Fold change < −2). While DSM17938 treatment only upregulated 28 genes including Gm49450, Lars2, Hist2h2aa2, etc., (Fold change > 2) and downregulated 13 genes including S100g, Akp3, Fabp6, etc., (Fold change < −2) (Figure 8C).

Next, these differentially expressed genes were subjected to KEGG pathway enrichment analysis. MFN041 group inhibited the pathways related to autoimmune and allergic diseases, such as asthma, autoimmune thyroid disease and systemic lupus erythematosus. On the contrary, it activated retinol metabolism, steroid hormone biosynthesis, linoleic acid metabolism, PPAR signaling pathway and other pathways (Figure 8D).

### Intervention mode I activated immune-related pathways and inhibited inflammation-related pathways

**FIGURE 9:**
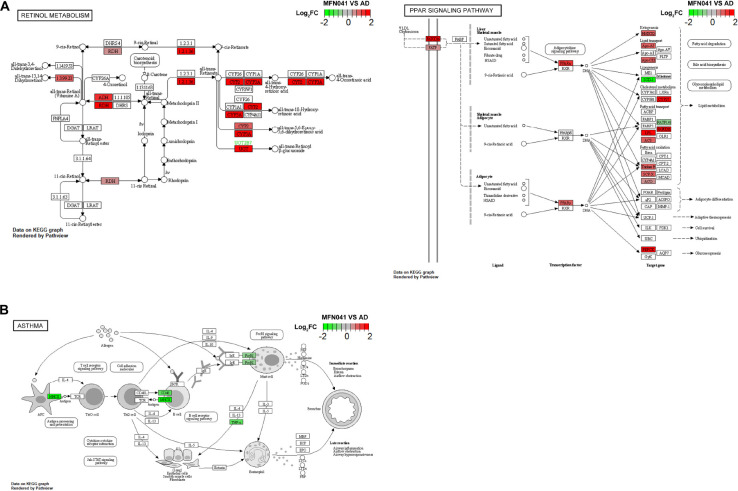
**(A)** KEGG pathway graph of retinol metabolism and PPAR signaling pathway, **(B)** asthma rendered by Pathview (*n* = 3), red for activation, green for inhibition. CON, normal control mice supplemented with sanitary saline; AD, supplementation with sanitary saline after weaning of pups before AD induction; DSM17938, supplementation with DSM17938 after weaning of pups before AD induction; LGG, supplementation with LGG after weaning of pups before AD induction; FN041, supplementation with FN041 after weaning of pups before AD induction; MFN041, FN041 supplementation in dams during the late trimester and lactation period and after weaning of pups before AD induction.

The expression of several genes of the retinol metabolic pathway was activated in PPs of MFN041 group, including ethanol dehydrogenase (ADH), retinol dehydrogenase (RDH) and aldehyde dehydrogenase 1 family member A2 (ALDH1A2), which would enhance the production of retinoic acid. In addition, the PPAR-α signaling pathway was also activated after MFN041 treatment, and the expression of genes such as HMGCS2, Apo-AI, Apo-CIII, CYP27, LPL, and ACS were upregulated. The PPAR-α signaling pathway regulates T cell differentiation through *trans*-activation and/or interaction with other transcription factors thereby reducing the inflammatory response and regulating lipid metabolism such as fatty acid metabolism, bile acid synthesis and glycine phospholipids (Figure 9A). Significant downregulation of MHCII, CD40 and TNF-α expression in the asthma pathway, blocking acute inflammatory responses such as bronchospasm, edema and airflow obstruction (Figure 9B). Decreased MHCII, CD40, TNF-α, C2, and C7 in the systemic lupus erythematosus signaling pathway further downregulate MAC expression and reduces tissue damage and inflammatory response (Supplementary Figure 1).

## Discussion

This study showed that supplementation with FN041 by intervention mode I was more effective in preventing AD than supplementation with FN041 by intervention mode II. On the one hand, mechanisms of FN041 supplementation to prevent AD by intervention mode I include enhancing the ileal mucosal barrier in pups, increasing the proportion of CD4^+^CD25^+^Foxp3^+^ Tregs in the spleen, promoting a balanced Th1/Th2 immune response, and remodeling the intestinal microbiota. On the other hand, it also activated retinol metabolism, the PPAR signaling pathway and downregulated pathways associated with allergy and autoimmune diseases such as asthma, autoimmune thyroid disease and systemic lupus erythematosus. Intervention mode II increased the number of CD4^+^CD25^+^Foxp3^+^ Tregs in the spleen and strengthened the intestinal mucosal barrier. However, the effect of reducing AD was much smaller than in intervention mode I.

In this study, AD symptoms were induced in mice by applying MC903 plus OVA to mouse ears. This approach greatly reduced the time required for modeling, and the mice showed signs of redness, itching, dryness, flaking and local erosions in the ears, which may be more like symptoms of AD in infants (18). However, this method may cause severe diarrhea and weight loss in pups (unpublished data), so more research needs to be done to find more appropriate doses of OVA (25).

We found that compared with the model group, FN041 supplementation by intervention mode I can significantly inhibit the ear symptoms of AD mice. Our previous study confirmed that FN041 ingested by maternal mice increased the relative abundance of *Limosilactobacillus reuteri* in breast milk, indicating that FN041 ingested by female mice can reach the intestinal tract of offspring through vertical transmission during lactation (7). However, the relative abundance of *Limosilactobacillus reuteri* in the ileum of mice supplemented with FN041 by intervention mode II did not increase, indicating that the relative abundance in the ileum of weaned mice could not be increased by gavage of FN041 directly. This may be because FN041 is a typical sIgA-coated bacterium. FN041 may promote the interaction between sIgA and intestinal flora in breast milk, thus mediating immune development (26).

Studies have shown that intestinal dysbiosis is closely associated with allergic diseases (27, 28). The supplementation of FN041 by intervention mode I increased the abundance of not only *Limosilactobacillus reuteri* but also *Akkermansia*, *Bifidobacterium and Faecalibacterium* in the ileum of the pups compared to the model group. The expression of ileal tight junction protein ZO-1 and the corresponding increase in the ratio of villi height to intestinal crypt depth attenuated intestinal mucosal injury in AD mice (29). Studies have shown that *Akkermansia* is associated with alterations in functional genes for host immune development. *Akkermansia* can stimulate IL-10 production, which induces Treg production (30). *Bifidobacterium* can help alleviate AD by inhibiting polarized Th2 immune responses, modulating gut microbiota and short-chain fatty acid metabolism (31). *Faecalibacterium* is the main butyric acid-producing bacteria found in the gut. Butyrate is one of the primary energy sources for colon cells, maintaining the integrity of the gut lining and preventing pathogens from entering the body through the gut. It stimulates villus growth and promotes mucin production. Furthermore, butyrate enhances the colonic barrier by increasing claudin synthesis and antimicrobial peptide production (32, 33). In the FN041 supplementation by intervention mode II group, the expression of ZO-1 and the number of Treg cells were increased, and the IgE level and ear thickness were decreased compared with the model group. In addition, there was no increase in the abundance of *Limosilactobacillus reuteri* in the ileum, but an increase in the abundance of *Lactobacillus Johnsonii*. One study found that *Lactobacillus Johnsonii* stimulated Caco-2 cells *via* the Toll-Like receptor 9 and thus improved the immune response (34).

Peyer’s patches transcriptome results showed that pups that acquired FN041 by intervention mode I activated retinol metabolism, the PPAR signaling pathway and inhibited asthma, type I diabetes mellitus, systemic lupus erythematosus and autoimmune thyroid disease pathways. Retinol is one of the forms of vitamin A. In this study, the retinol metabolic pathway was activated by vertical transmission of FN041, which would lead to retinoic acid (RA) production. It was found that children with allergic diseases had elevated Treg and CD27^+^ IgA^+^ B cells to compensate for chronic inflammation (35). All these cells are required for RA induction and therefore may contribute to retinal degeneration. In this study, vertical transmission of acquired FN041 to promote retinol metabolism may be the key to preventing AD inflammation. In contrast, it has been established that RA both encourages the growth of thymocytes and controls the process of apoptosis (36, 37). Endogenous retinoid synthesis and glucocorticoid-like retinoids may actually control thymic proliferation and selection by being present in the thymus in enough amounts to be useful (38). Members of the nuclear receptor superfamily for steroid hormones, PPARs are crucial for controlling lipid metabolism, energy homeostasis, atherosclerosis and blood sugar levels. Recent studies have shown that they play an important role in the regulation of inflammation (39). Vertical transmission of FN041 processing activates the PPAR-α pathway, and a study confirmed the use of PPAR-α agonists for the treatment of autoimmune diseases. PPAR-α agonists specifically attenuate IL-6-induced APR *in vitro* and *in vivo* by downregulating the hepatic expression levels of SAA, HG, FIB-α, -β and -γ (40). PPAR-α agonists such as gemfibrozil and fenofibrate increase the production of the Th2-type cytokine IL-4, inhibit the proliferation of myelin basic protein AC1-11-specific TCRT cells and decrease NO production by microglia (41). The clinical symptoms of experimental autoimmune encephalomyelitis are suppressed by oral treatment of gemfibrozil and fenofibrate. More significantly, gemfibrozil affected the release of cytokines from human T-cell lines by decreasing interferon and raising IL-4. These results suggest that PPAR-α agonists may be attractive candidates for use in human inflammatory diseases such as multiple sclerosis. In addition, asthma is closely associated with the development of atopic dermatitis. Atopic dermatitis, if not well controlled early in life, may trigger asthma later in life (42). FN041 by intervention mode I significantly inhibits the asthma pathway, blocks acute inflammatory responses such as bronchospasm, edema and airflow obstruction, and inhibits the systemic lupus erythematosus inflammatory signaling pathway, reducing tissue damage and inflammatory response.

The lactation period is a critical window for immune development in pups, during which the gut microbiota shows more significant flexibility and plasticity compared to adults (43). Therefore, our next step will be to validate the effect of FN041 supplementation in newborn mice for AD prevention. Furthermore, LGG has been shown to regulate intestinal flora, improve immunity, and has a preventive effect on atopic dermatitis (44, 45). DSM17938 and FN041 are both *Limosilactobacillus reuteri* isolated from breast milk. DSM17938 has been demonstrated to relieve diarrhea and prevent and relieve allergies (46, 47). In this study, FN041 showed a better AD prevention effect than DSM17938 and LGG intervention. The exact mechanism needs to be further investigated. This may be due to the better adherence of FN041 (unpublished data). The addition of DSM17938 to neonatal formula promoted growth in children and was well-tolerated (48, 49), implying that FN041 could be used in the maternal formula and infant formula during the late trimester and lactation for the prevention of neonatal AD.

FN041 was isolated from breast milk in southern Gansu Province, China. Tibetans in southern Gansu have traditionally lived on farming and raising livestock, and the incidence of allergic diseases is significantly lower than in urban areas. The relative abundance of *Limosilactobacillus reuteri* in the breast milk microbiota in Tibetans is much higher than in the urban population (50). It may be one of the typical strains that lost in the process of industrialization, which may provide a new idea for the prevention and treatment of AD in the future.

## Conclusion

The main contribution of this study is that compared with direct supplementation of FN041 after weaning, vertical transmission of FN041 to the gut of offspring is more effective in preventing AD. On the one hand, the vertical transmission of FN041 into the intestine of pups could regulate the ileum microbiota, strengthen the ileal mucosal barrier and increase the number of Treg in the spleen. On the other hand, oral tolerance-related pathways of PPs were activated, including the retinol metabolism and PPAR signaling pathway, while immune disease and allergy-related pathways are inhibited including asthma. Therefore, this study demonstrates that prenatal and postnatal administration of FN041 is an effective way to prevent AD in offspring, and its mechanism is related to remodeling of ileal microbiota and PPs immune response. This study provides a reference for the application strategy of breast milk derived probiotics to prevent infant AD and shows the feasibility of FN041 to prevent other allergic diseases.

## Data availability statement

The original contributions presented in the study are publicly available. All raw sequencing data were deposited at the NCBI Sequence Read Archive with accession number PRJNA827499.

## Ethics statement

The animal study was reviewed and approved by the Qingdao University Animal Ethics Committee.

## Author contributions

JZ: data analysis, visualization, and writing – original draft. GX: data curation and writing – original draft. XL, HT, and HL: experimental measurements. HC and JC: funding acquisition. CQ, RY, and JS: study designing and writing – review and editing. All authors contributed to the article and approved the submitted version.
