# Supplementary figures and images for: Limosilactobacillus reuteri FN041 prevents atopic dermatitis in pup mice by remodeling the ileal microbiota and regulating gene expression in Peyer’s patches after vertical transmission
Source: Front Nutr. 2022 Sep 28;9:987400. doi: 10.3389/fnut.2022.987400 (PMC9554658; doi:10.3389/fnut.2022.987400)

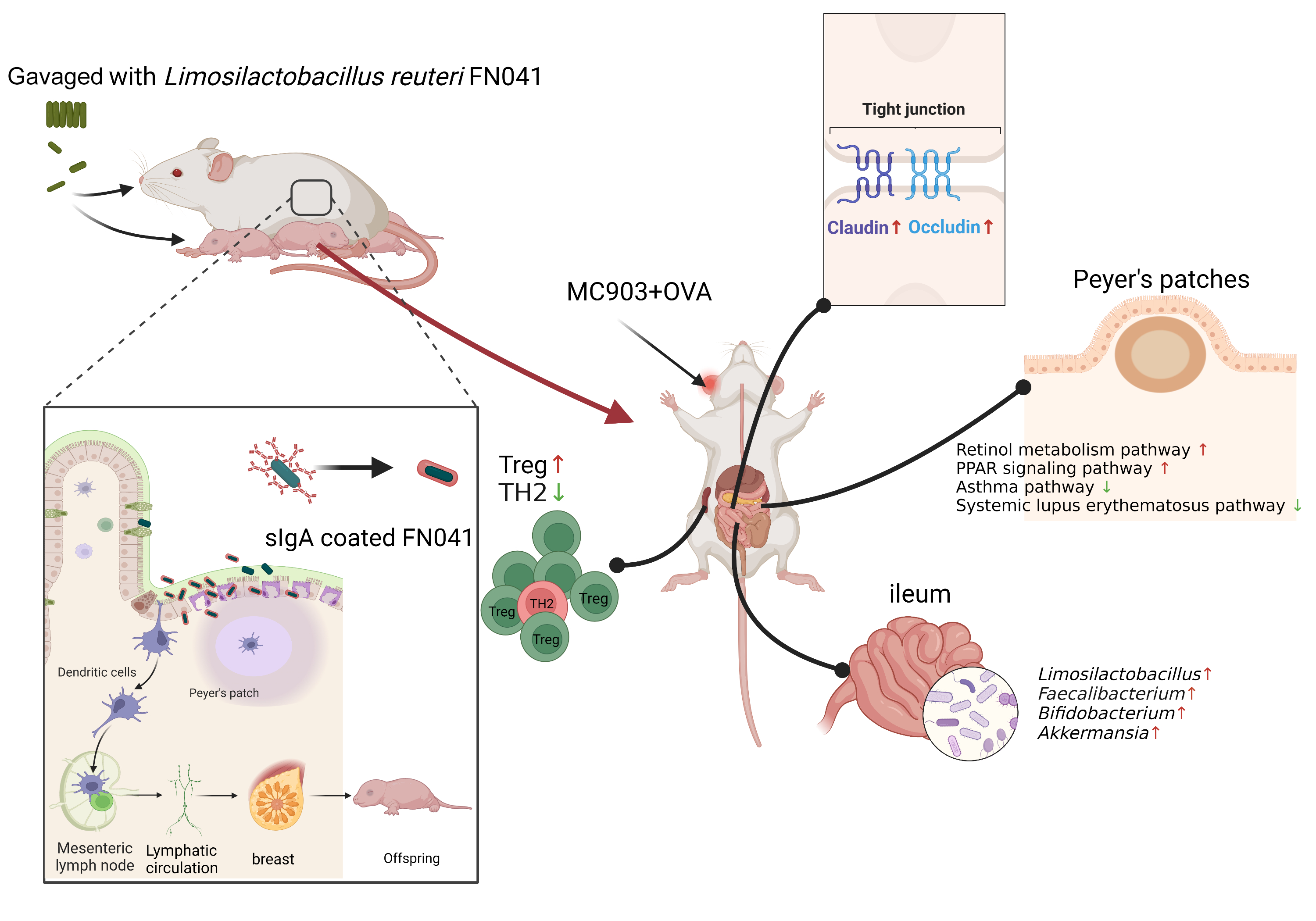

Supplement: Supplementary file 1 [file Image_1.TIF]
