# Supplementary material for: Limosilactobacillus reuteri FN041 prevents atopic dermatitis in pup mice by remodeling the ileal microbiota and regulating gene expression in Peyer’s patches after vertical transmission
Source: Front Nutr. 2022 Sep 28;9:987400. doi: 10.3389/fnut.2022.987400 (PMC9554658; doi:10.3389/fnut.2022.987400)
Supplement: Supplementary file 2 [file Table_1.docx]

**Supplementary materials**

**Supplementary methods**

16S rDNA gene sequencing

To maximize the effective length of the MiSeq 250/300PE sequencing reads, a region of approximately 469 bp containing the highly variable V3 and V4 regions of the 16S rRNA genes was targeted for sequencing using PCR primers: forward 5'- CCT ACG GRR BGC ASC AGK VRV GAA T -3' and reverse 5'- GGA CTA CNV GGG TWT CTA ATC C -3'. In addition, an index linker was added to the end of the 16S rDNA PCR product for next-generation sequencing (Illumina, San Diego, CA, USA). The first-round PCR product was used as a template for the second round PCR amplicon enrichment (94°C for 3 min, followed by 24 cycles at 94°C for 5 s, 57°C for 90 s and 72°C for 10 s, and a final extension at 72°C for 5 min). PCR reactions were performed in triplicate using a 25 μL mixture containing 2.5 μL TransStart Buffer, 2 μL dNTPs, 1 μL of each primer, and 20 ng of template DNA. DNA library concentrations were verified using a Qubit 3.0 fluorometer. Libraries were quantified to 10 nM and subsequently multiplexed and loaded on an Illumina MiSeq instrument (Illumina, San Diego, CA, USA) according to the manufacturer's instructions. Sequencing was performed using PE250/300 paired ends; image analysis and base calling were performed using the MiSeq control software embedded in the MiSeq instrument.

All raw sequencing data were deposited at the NCBI Sequence Read Archive with accession number PRJNA827499.

Histological Examination

Mice ear skin was fixed with 4% phosphate-buffered paraformaldehyde. The tissue was stained with H&E to assess inflammatory cell infiltration. Infiltration of eosinophils and mast cells was detected with Carbol 2R hematoxylin and toluidine blue, respectively. The number of eosinophils and mast cells is the number of cells in 10 random fields of view of mice ear sections. Mice colon samples were removed and fixed in 10% buffered formalin, embedded in paraffin, and cut into 5 mm thick sections. Briefly, sections were deparaffinized and washed in PBS, soaked in 3% H_2_O_2_ for 10 min, and then antigen retrieval was washed in PBS and incubated with goat serum albumin for 20 min. Sections were then incubated with rabbit anti-zonulin-1(1:100, BS-1329R, Bios, Beijing, China) at 37℃ for 2.5 h. The embedded tissues were washed with PBS. After the sections were incubated with biotinylated anti-rabbit IgG and then processed by the S-A/HRP, color was developed in the diaminobenzidine (DAB) substrate solution. The sections were then counterstained with hematoxylin, dehydrated, cleared, and permanently mounted. Villi height and crypt depth of mice colon were calculated from 10 random fields of view. The sections were observed under the Olympus BX51microscope (Olympus Optical Company, Shanghai, China).

AD severity scoring method

The degree of AD symptoms was observed on days 2, 4, 6, 8, 10 of AD modeling and scored as follows:

(1) Number of scratching bouts (per minute): 10 points for >80 times, 7 points for 50-80 times, 4 points for 20-50 times, 1 point for 10-20 times, 0 points for <10 times.

(2) Erythema/hemorrhage in the ear: 10 points for an area greater than 80% of the total skin area of the ear; 7 points for an area between 50% and 80%; 4 points for an area between 20% and 50%; 1 point for an area less than 20%; 0 points for no erythema/hemorrhage.

(3) Eruption of the ear: 10 points for an area of eruption greater than 80% of the total skin area of the ear; 7 points for an area between 50% and 80%; 4 points for an area between 20% and 50%; 1 point for an area less than 20%; 0 points for no symptoms of eruption.

(4) Ear edema: 10 points for dark red, uneven and obvious swelling of the ears; 7 points for light red, uneven and weak edema of the ears; 4 points for the slightly red and uneven surface of the ears; 1 point for smooth ears close to the skin color of mice's ears, and 0 point for the absence of the above symptoms.

(5) Scaling-like changes in the ear: 10 points for an area greater than 80% of the total skin area of the ear; 7 points for an area between 50% and 80%; 4 points for an area between 20% and 50%; 1 point for an area less than 20%; 0 points for no scaling-like changes.

The total score was the sum of 5 items/5.

RNA sequencing for Peyer’s patches

RNA was extracted from pooled Peyer’s patches using RNeasy kit and the concentration and purity were measured with NanoDrop 2000 Spectrophotometer (Thermo Fisher Scientific, Wilmington, USA). RNA integrity was assessed using the RNA Nano 6000 Assay Kit of Agilent Bioanalyzer 2100 system (Agilent Technologies, Santa Clara, USA). High-quality RNA was used for cDNA libraries construction and sequencing at Biomarker Technologies Corporation (Biomarker Technologies Corporation, Beijing, China). The RNA-Seq libraries were constructed according to NEBNext UltraTM RNA Library Prep Kit for Illumina (New England Biolabs, Ipswich, USA) following manufacturer’s recommendations. Briefly, mRNA was purified by NEBNext Poly (A) mRNA Magnetic Isolation Module. The isolated mRNA was fragmented and used to synthesize the first cDNA. Second strand cDNA synthesis was generated using DNA Polymerase I and RNase H. The double-stranded cDNAs were purified by Agencourt AMPure XP system (Beckman Coulter, Brea, USA) and subjected to end repair and adapter ligation. The ligation products were enriched by PCR amplification and purified using Agencourt AMPure XP system. Sequencing reactions were carried out on the Illumina HiSeq 2500.

The raw reads were firstly processed through in-house perl scripts. Clean reads were obtained by removing reads containing adapter sequences, unknown nucleotides> 5%, low quality reads. The clean reads were mapped to mouse genome (mm10) with TopHat239. Gene expression levels were estimated using fragments per kilobase of exon per million fragments mapped (FPKM).

**Supplemental Figure**


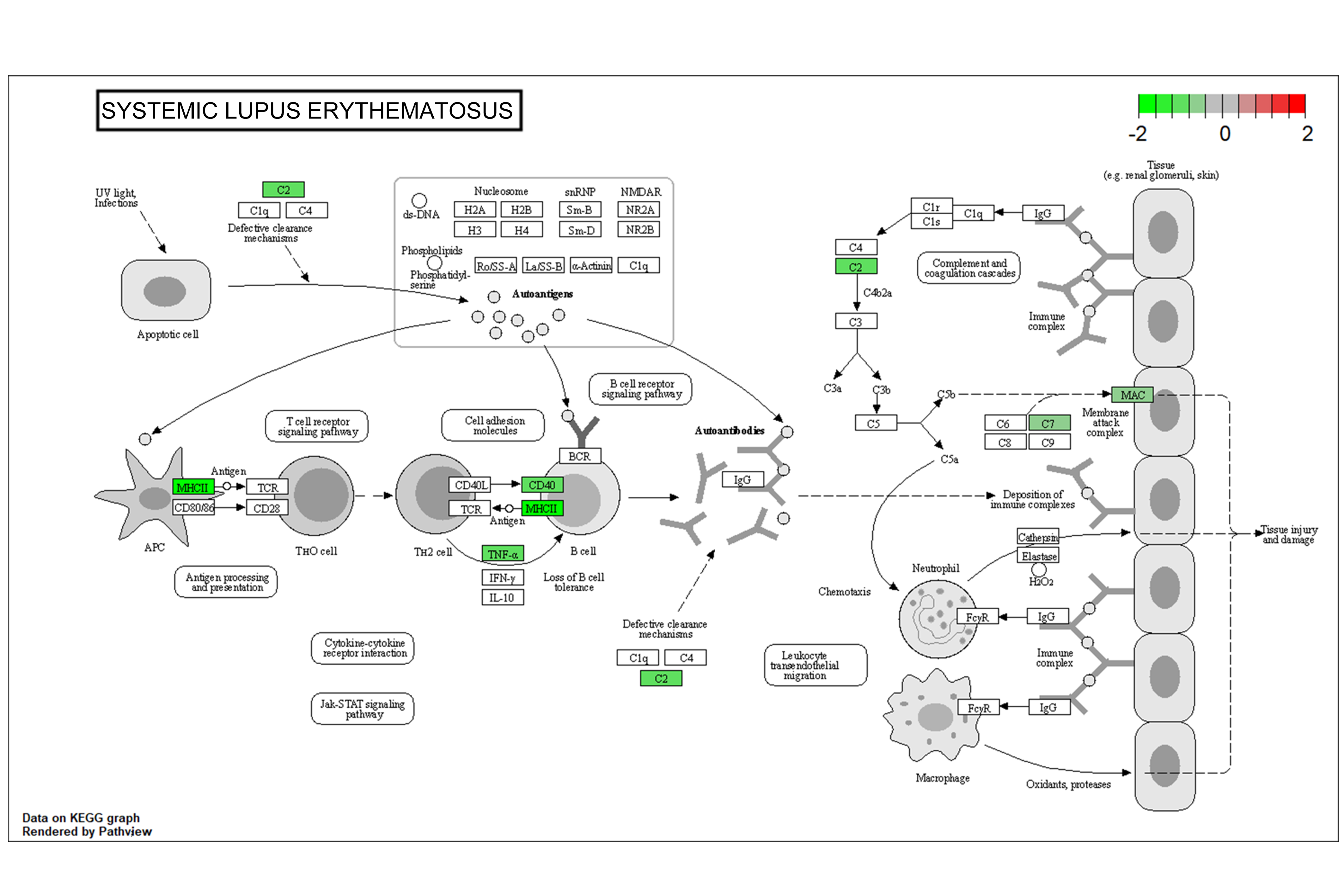


Figure S1. MFN041 treatment inhibited the systemic lupus erythematosus pathway rendered by Pathview.
